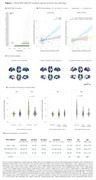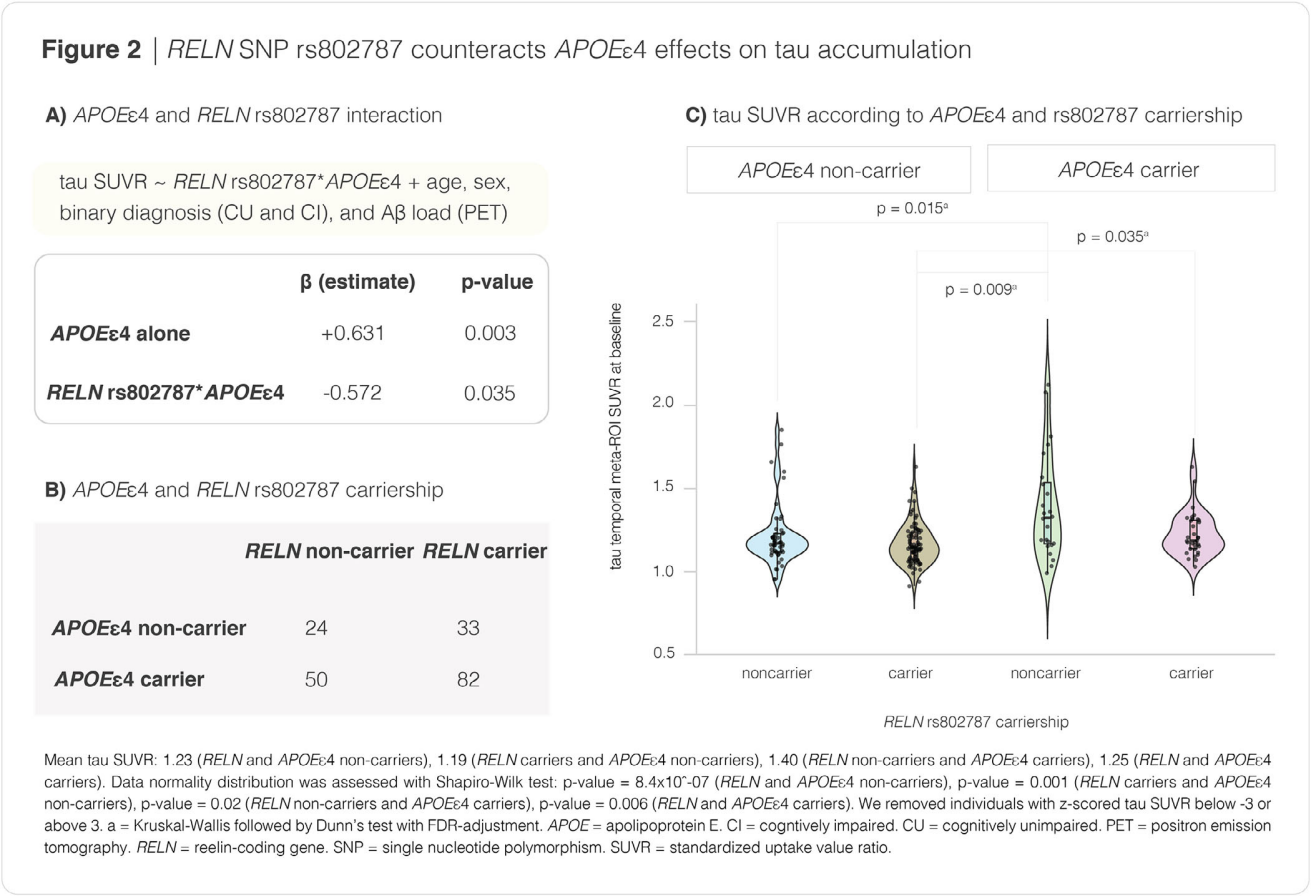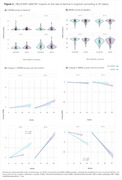# *RELN* SNP rs802787 protects against Aβ‐driven tau pathology, counteracts *APOE*ε4 effects, and slows cognitive decline in sporadic late‐onset Alzheimer's disease

**DOI:** 10.1002/alz70855_106073

**Published:** 2025-12-24

**Authors:** Giovanna Carello‐Collar, João Pedro Ferrari‐Souza, Marco Antônio De Bastiani, Thomas Hugentobler Schlickmann, Christian Limberger, Guilherme Povala, Wyllians Vendramini Borelli, Tharick A Pascoal, Pedro Rosa‐Neto, Diogo O. Souza, Eduardo R. Zimmer

**Affiliations:** ^1^ Universidade Federal do Rio Grande do Sul, Porto Alegre, RS, Brazil; ^2^ Universidade Federal do Rio Grande do Sul, Porto Alegre, Rio Grande do Sul, Brazil; ^3^ University of Pittsburgh, Pittsburgh, PA, USA; ^4^ McGill University, Montreal, QC, Canada

## Abstract

**Background:**

A rare reelin gene variant (*RELN*‐COLBOS mutation) delayed dementia onset by 30 years in an autosomal dominant Alzheimer's disease (ADAD) mutation carrier. Despite a high amyloid‐β (Aβ) load, the brain had low tau accumulation, suggesting that this mutation conferred resilience against tau pathology and cognitive decline. However, whether *RELN* variants protect against sporadic late‐onset Alzheimer's disease (LOAD) remains unknown. Here, we evaluated the impact of *RELN* single nucleotide polymorphisms (SNPs) on AD pathophysiology and cognitive decline in LOAD.

**Method:**

We included 189 individuals from ADNI with available data on *RELN* SNPs, Aβ‐ and tau‐PET, CSF Aβ_1‐42_ and ptau_181_, *APOE*ε4 status, neuropsychological tests (CDRSB and MMSE), and clinical diagnosis. We investigated the impact of *RELN* carriership on the association between Aβ and tau burden through regional‐ and voxel‐wise linear regressions, and on cognitive decline based on AT biomarker profile with a linear mixed‐effect model. We also analyzed the interaction effects between *RELN* and *APOE*ε4 carriership on tau pathology (Bonferroni's adjusted *p*‐value < 0.05).

**Result:**

Of the *RELN* SNPs available in ADNI (Figure 1A), *RELN* rs802787 protected against Aβ‐driven tau pathology (β = ‐0.603, adj. *p*‐value = 0.0002; Figure 1B). At the voxel level, this protection was mostly associated with the temporal lobe (Figure 1C). Also, *RELN* rs802787 carriers exhibited a reduced *APOE*ε4‐related tau burden (β = ‐0.572, *p*‐value = 0.035; Figure 2). Stratifying individuals by AT status revealed that *RELN* rs802787 carriership did not affect cognitive decline in Aβ‐ groups (Figure 3). In contrast, A+T‐ carriers showed a slower change in CDRSB (β = ‐0.45, *p*‐value = 0.007) and MMSE (β = +0.3, *p*‐value = 0.001) scores over the months, an effect absent in individuals with high tau burden (A+T+; Figure 3).

**Conclusion:**

Our findings suggest that *RELN* rs802787 confers resilience against Aβ‐driven tau pathology and cognitive deterioration in LOAD. The reduced *APOE*ε4‐associated tau burden suggests a potential mechanism by which *RELN* could modulate tau accumulation. To our knowledge, this is the first *RELN* variant identified as protective in LOAD. Our results suggest that reelin signaling is an important player in AD pathophysiology, underscoring it as a promising target for AD therapeutics.